# Sexual Orientation in Individuals With Congenital Adrenal Hyperplasia: A Systematic Review

**DOI:** 10.3389/fnbeh.2020.00038

**Published:** 2020-03-13

**Authors:** Elisabeth Daae, Kristin Billaud Feragen, Anne Waehre, Ingrid Nermoen, Henrik Falhammar

**Affiliations:** ^1^Oslo University, Oslo, Norway; ^2^Akershus University Hospital, Lillestrøm, Norway; ^3^Karolinska Institute, Karolinska University Hospital, Stockholm, Sweden

**Keywords:** 21-hydroxylase deficiency, partnership, homosexuality, bisexuality, androgen effects

## Abstract

Congenital adrenal hyperplasia (CAH) is a genetic condition of the steroidogenic enzymes in the adrenal cortex normally leading to variable degrees of cortisol and aldosterone deficiency as well as androgen excess. Exposure to androgens prenatally might lead to ambiguous genitalia. The fetal brain develops in traditional male direction through a direct action of androgens on the developing nerve cells, or in the traditional female direction in the absence of androgens. This may indicate that sexual development, including sexual orientation, are programmed into our brain structures prenatally. The objective of this study was to perform a systematic review of the literature, investigating sexual orientation in individuals with CAH. The study also aimed at identifying which measures are used to define sexual orientation across studies. The review is based on articles identified through a comprehensive search of the OVIDMedline, PsycINFO, CINAHL, and Web of Science databases published up to May 2019. All peer-reviewed articles investigating sexual orientation in people with CAH were included. Quantitative, qualitative, and mixed methods were considered, as well as self-, parent-, and third-party reports, and no age or language restrictions were enforced on publications. The present review included 30 studies investigating sexual orientation in patients with CAH assigned female at birth (46, XX) (*n* = 927) or assigned male at birth (46, XY and 46, XX) (*n* = 274). Results indicate that assigned females at birth (46, XX) with CAH had a greater likelihood to not have an exclusively heterosexual orientation than females from the general population, whereas no assigned males at birth (46, XY or 46, XX) with CAH identified themselves as non-heterosexual. There was a wide diversity in measures used and a preference for unvalidated and self-constructed interviews. Hence, the results need to be interpreted with caution. Methodological weaknesses might have led to non-heterosexual orientation being overestimated or underestimated. The methodological challenges identified by this review should be further investigated in future studies.

## Introduction

The first known patient with congenital adrenal hyperplasia (CAH) was described in 1865 by Luigi De Crecchio, a professor of anatomy in Napoli (Delle Piane et al., 2015). When performing an autopsy of a man named Giuseppe Marzo, who died suddenly in his forties, the professor expressed marked surprise at the findings of a uterus and fallopian tubes in this man, who also had enlarged adrenals. The patient had a six centimeter long penis but no testes. At birth, he was regarded a female, but at 4 years of age, he was reconsidered a male. As an adult, he fell in love with a girl; however, when he proposed to marry her, she ran away after realizing he was called Giuseppina in his birth certificate (Delle Piane et al., 2015). Giuseppe probably died of a salt-wasting crisis, even if the story goes that he died of a broken heart (New, 2011). CAH is a common cause of disorders of sex development (DSD), and Guiseppe's story is still relevant today.

CAH is a genetic condition of the steroidogenic enzymes in the adrenal cortex. The majority, 95–99% are caused by 21-hydroxylase deficiency (21OHD), in which gives impaired cortisol and varied degrees of aldosterone production, accompanied by adrenocorticotropic hormone (ACTH)-driven increase in, adrenal androgens and steroid precursors (Arlt et al., 2010; Gidlof et al., 2013; Speiser et al., 2018). Traditionally, classical CAH is divided into salt wasting (SW) and simple virilizing (SV), based on the severity of aldosterone deficiency (El-Maouche et al., 2017). Non-classic (NC) CAH is a mild variant often diagnosed late, if ever. The androgen levels are only mildly elevated and no apparent cortisol deficiency is present. In some females clitoral hypertrophy may be present but usually only mild signs of hyperandrogenism can be found (e.g., hirsutism, acne, and oligomenorrhea) (Nordenstrom and Falhammar, 2018). 46, XX individuals with classical CAH are born with a variable degree of external genital ambiguity owing to exposure of excess androgens prenatally (Nordenskjold et al., 2008; Almasri et al., 2018), and is one of the most common causes of ambiguous genitals in 46, XX. In addition, some of the rarer CAH variants, such as 11β-hydroxylase deficiency (46, XX), 3β-hydroxysteroid dehydrogenase type 2 deficiency (46, XY), P450 oxidoreductase deficiency (both genders), and lipoid adrenal hyperplasia or SCC enzyme deficiency, can result in atypical genitalia (El-Maouche et al., 2017; Bulsari et al., 2018; Al Alawi et al., 2019). To restore typical genital appearance and function in those most severely affected by CAH and reared as girls, the standard of care has been early genital reconstructive surgery (Almasri et al., 2018). However, genital surgery raises questions and criticism regarding its indications, timing, and choice of surgical techniques (Wolffenbuttel and Crouch, 2014; Mouriquand et al., 2016).

Since, the introduction of replacement therapy with glucocorticoids and mineralocorticoids in the 1950s, patients with CAH usually survive into adulthood (Bartter et al., 1950; Falhammar and Thoren, 2012; El-Maouche et al., 2017). However, the glucocorticoid doses are usually supraphysiological which may partly explain the increased cardiometabolic morbidity and increased mortality seen in adults (Falhammar et al., 2014a, 2015). Despite postnatal treatment, females with CAH show altered play behavior (Hines, 2004a). They tend to prefer rough and tumble play, masculine hobbies, typical boys' toys, and males as playmates, and have some male-typical personality features (Hines et al., 2004; Meyer-Bahlburg et al., 2004; Frisen et al., 2009; Hines, 2011a; Pasterski et al., 2011, 2015).

Androgen levels may also influence psychosexual development, including sexual orientation (Meyer-Bahlburg, 1984; Hines et al., 2004; Gooren, 2006; Balthazart, 2011; Bailey et al., 2016). Sexual orientation is a multidimensional concept, and includes three components: attraction (physiological response, sexual or romantic desires, and attachments), behavior (sexual activities), and identity (self-chosen labels such as “gay/lesbian,” “bisexual,” “heterosexual,” or “straight”; Savin-Williams, 2006; Wolff et al., 2017). Rates of homosexuality vary across time, cultures, age groups, and sexes, and the numbers also vary depending on the component being assessed (Savin-Williams, 2006). Determining the accurate numbers of non-heterosexual individuals in any given population can be difficult because the numbers depend upon the methods and assessment used (Savin-Williams, 2006). Studies indicate that ~3–6% of males and 1–4% of females in the general population display predominantly homosexual attractions (Diamond, 1993; Savin-Williams, 2009; Savin-Williams et al., 2012). However, according to Kinsey et al.'s (1948) findings, nearly half (46%) of the general population reported engaging in both heterosexual and homosexual activities, or reacting to people of both sexes, in the course of their adult lives. Thus, bisexual orientation could be considered almost as common as heterosexuality.

Androgens and estrogens are involved in the organizational phase of the brain *in utero*, creating sex differences in regions including the hypothalamus, septum, preoptic area, and amygdala, areas linked to emotional reactions, behavior, learning, and endocrine regulation (Reinisch and Sanders, 1992; Hines, 2004b; Baum, 2006; Gooren, 2006). Animal studies have shown that prenatal and early postnatal sex-atypical levels of androgens or estrogens (derived by aromatization of androgens within brain cells) have an organizational effect on the developing nervous system. In 1959, it was reported that adult female guinea pigs exposed to androgens during gestation showed less feminine copulatory behavior and more masculine behavior (Phoenix et al., 1959). The novelty was the concept that the brain had been masculinized, meaning that steroid hormones could alter the development of the nervous system. The data from these early studies became the background for the organizational-activational hypothesis (Phoenix et al., 1959; Young et al., 1964). According to this hypothesis, the androgens or their metabolites, acts on the brain of males to irreversibly alter the substrate mediating mating behavior (Phoenix et al., 1959). Later effects of androgens were on the other hand anticipated to “activate” the already masculinized substrate. These activational effects of gonadal hormones can occur at any time of life, but are predominantly studied in adults (McCarthy and Arnold, 2011). The hypothesis also claimed that the organizing actions of hormones occur at a specific period of sensitivity when masculine neural circuits develop or are maintained, and feminine circuits are prevented from developing or are allowed to atrophy. To test whether the effect of prenatal treatment with testosterone propionate applied to other species than guinea pigs, rhesus monkeys were investigated as they as non-human primates, and are more complex, social, and physiologically similar to humans than rodents (Young et al., 1964; Thornton et al., 2009). Studies verified that prenatal androgens have permanent effects in rhesus monkeys on the neural circuits underlying sexually dysmorphic behaviors, including sexual and social behaviors, both of which are also influenced by social experience (Thornton et al., 2009). The rhesus monkeys do not rely upon estrogenic metabolites of androgens for full masculinization (unlike most rodent species, and like guinea pigs) or defeminization (unlike any other species studied), thus making the monkey the only species that obviously appear to rely solely upon androgens for hormonal organization of the nervous system (Thornton et al., 2009; Wallen, 2009). Similarly, males deprived of early androgen exposure through castration or anti-androgen treatment fail to show normal male-typical neural characteristics and behavioral development, regardless of adult hormonal exposure (Goy and McEwen, 1980; Hines, 2004a). This hypothesis assumes that if a fetus is exposed to sex-atypical levels of androgens or estrogens, the exposure may alter the sex differentiation in a way that fosters sex-atypical behavior and partner choice. This results in sexual dysmorphic brain structure and behavior, supporting the development of masculine behavior and estrogens supporting the defeminization of behavior (Dominguez-Salazar et al., 2002; Hrabovszky and Hutson, 2002; Adkins-Regan, 2011; Henley et al., 2011). During the last two decades, advances in genetics have broadened the focus of research on sexual differentiation, to include several genes involved in the sexual differentiation of the gonads (Fleming and Vilain, 2005), and possibly, of the brain (Arnold, 2017). Research on the specific genetic mechanisms in the brain is still in its early days, but the sexual differentiation of the human brain is most likely a multifactorial process including both sex hormone and sex chromosome effects, acting in parallel or in combination (Bakker, 2019).

A number of human behaviors show reliable sex differences (Hines, 1998). These include sexual orientation, most men preferring women sexually and most women preferring men. The origins of sexual orientation are still not well-understood (Ngun and Vilain, 2014). Sexual orientation in humans could be influenced by biological factors (Swaab and Garcia-Falgueras, 2009), genetic factors (Fleming and Vilain, 2005), immune responses (Blanchard, 2001; Bogaert et al., 2018), chemical factors (Ellis and Cole-Harding, 2001; Ellis and Hellberg, 2005), social factors (Colapinto, 2001; Swaab, 2004), and/or hormones (Bao and Swaab, 2011). Hormonal differences as a causal hypothesis has been the most influential (Money and Ehrhardt, 1972; Dörner, 1976; Meyer-Bahlburg, 1984; Ellis and Ames, 1987; Zucker, 2005). However, the direct evidence for prenatal hormone influences on human adult sexual orientation remains sparse (Hines, 2011b; Jordan-Young, 2012; Hill et al., 2013). One way of studying this phenomenon in humans is to study individuals who prenatally developed in a non-typical hormone environment, such as individuals with CAH.

Several studies indicate that females with CAH may have increased rates of bi-/homosexuality as demonstrated in erotic/romantic fantasies/dreams and sexual attraction, and to a lesser degree in overt homosexual action (Ehrhardt et al., 1968a; Ehrhardt, 1979; Money et al., 1984; Dittmann et al., 1992; Frisen et al., 2009). In contrast, only a few studies have investigated sexual orientation in males with CAH (Falhammar et al., 2012). Not many studies have explored how (medical aspects of) living with CAH could potentially affect males psychologically (Daae et al., 2018). There are few studies on sexuality in males with CAH (Hines et al., 2004; Arlt et al., 2010; Dudzinska et al., 2014; Falhammar et al., 2014b), and more information is needed in order to identify potential challenges. No comprehensive summary of the literature on sexual orientation in both males and females with CAH currently exists. In 2018, Gondim et al. (2018) conducted what they defined as a descriptive review of sexual orientation in 46, XX patients with CAH. This review did not include males (46, XX or 46, XY), and little information is provided about which dimensions of sexual orientation were measured. The objective of the present study was therefore to perform a systematic review of the literature, investigating sexual orientation in individuals with CAH, including both genders, hence assigned females and males (46, XY and 46, XX). The current review also aimed at identifying measures used to define sexual orientation across studies.

## Methods

### Inclusion and Exclusion Criteria

A systematic review of the literature was performed, following the recommended procedure for systematic reviews and meta-analyses, the PRISMA statement (Moher et al., 2015). All original peer-reviewed articles, published before mid-May 2019 and investigating sexual orientation in individuals with CAH were included. Quantitative, qualitative, and mixed methods investigations were considered. No age or language restrictions were imposed on the samples, and all methods of measurement (self-, parent-, and third-party reports) were included. Excluded were unpublished dissertations, case reports, review articles, editorials, and meeting abstracts. Studies on disorders of sex development (DSD) as a group, where results on CAH were not provided separately, as well as studies considering only the impact of surgery or gender identity were excluded. Articles with <10 subjects were excluded to minimize the risk of selection bias.

### Search Strategy

The OVID Medline, PsycINFO, CINAHL, and Web of Science databases were first systematically searched by the first author (ED) and discussed with the second author (KBF). The following search terms were used pertaining to diagnostic and psychological keywords: congenital adrenal hyperplasia, 21-hydroxylase deficiency, sexual orientation, gender identity, sexual identity, gender dysphoria, and gender incongruence. The Boolean operator OR was used between search terms within diagnostic concepts, and within search terms covering sexual orientation, while AND was used between these two categories (diagnostic terminology and concepts of sexual orientation). The search strategy had no filters. Reference lists of all included full-text articles were searched, to identify any potential additional relevant studies that had not yet been identified (*n* = 6).

### Assessment of Methodological Quality

**Figure 1:**
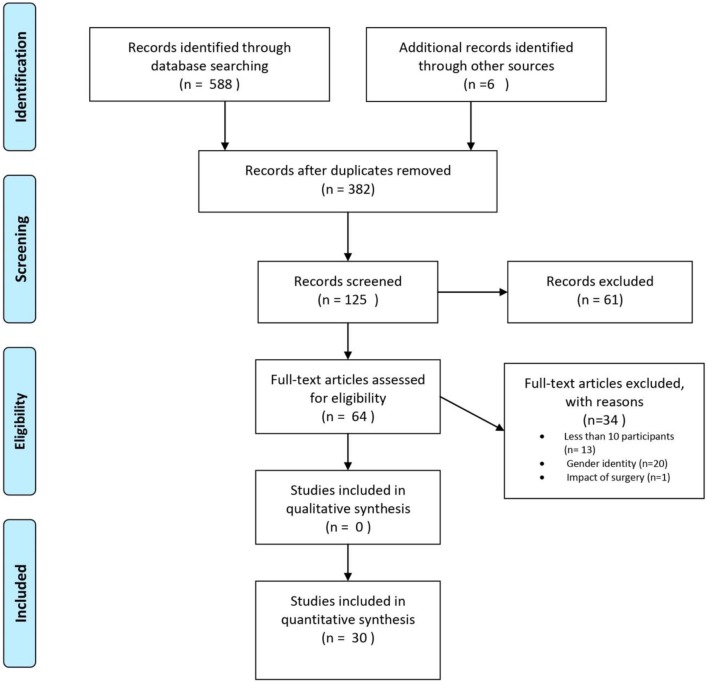
PRISMA flow chart, illustrating the procedure for article inclusion and exclusion in a systematic review of sexual orientation in individuals with congenital adrenal hyperplasia. From Moher et al. (2009).

The systematic search initially yielded 588 articles. After the search, all identified articles were merged using EndNote X9 for reference management, and duplicates were removed. After removing duplicates, 382 articles remained. Articles were first screened by title for relevance and then by abstract. A total of 64 articles were selected for full-text reading, from which 30 studies were included in the present systematic review (Figure 1). The first and second author (ED and KBF) independently screened titles and abstracts for eligibility, in order to select potential articles for full-text reading. The lists were compared and discussed, in order to check for consensus. There was no disagreement between the two authors, but uncertainties were discussed with the last author (HF), so that full consensus was obtained. In two cases, correspondence with author(s) of the included papers was necessary to clarify unclear aspects necessary when reporting results, such as information about outcome measures or unclear results. Questions used to assess the inclusion or exclusion of articles after full-text reading can be found in the Appendix. A total of 34 articles were excluded due to <10 participants (*n* = 13), data on gender identity only (*n* = 20), and impact of genital surgery (*n* = 1).

### Data Extraction

Two of the authors (ED and KBF) independently collected information about authors, outcome measures, countries, age range, and methods/design, using a data extraction form (see Appendix) made by one of the authors (ED). The data are presented using the same terminology, definitions, and interpretations as used in the original articles.

## Results

### Sexual Orientation in Individuals With CAH

A total of 30 articles assessing sexual orientation in individuals with CAH were included in the systematic review, as can be seen in Table 1 (Ehrhardt et al., 1968a; Lev-Ran, 1974; Ehrhardt, 1979; Money et al., 1984; Ellis and Ames, 1987; Mulaikal et al., 1987; Slijper et al., 1992; Kuhnle et al., 1993; May et al., 1996; Zucker et al., 1996; Dittmann, 1997; Hines et al., 2004; Morgan et al., 2005; Gupta et al., 2006; Johannsen et al., 2006; Brinkmann et al., 2007; Gastaud et al., 2007; Liang et al., 2008; Meyer-Bahlburg et al., 2008; Frisen et al., 2009; Lee et al., 2010; Fagerholm et al., 2011; Falhammar et al., 2012, 2017; Jurgensen et al., 2013; Lesma et al., 2014; Kanhere et al., 2015; Binet et al., 2016; Callens et al., 2016; Khorashad et al., 2017). Four of these studies explored sexual orientation in males with CAH (Hines et al., 2004; Lee et al., 2010; Falhammar et al., 2012, 2017). Sample sizes ranged from 11 to 221 individuals with CAH. Females were defined as 46, XX and males as 46, XY, unless stated otherwise.

**Table 1:** Overview and details of the articles included in the present systematic review.

**Reference**	**Sample size** **(CAH)**	**Age range (years)**	**CAH-type**	**Karyotype**	**Prader grade**	**Gender of rearing**	**Informants**	**Country**	**Comparison group**	**Study design**
Binet et al. (2016)	Females (*n =* 28)	16–40	SW (*n =* 4) SV (*n =* 24)	46, XX	II–IV	Unknown	Patients and parents	France	Three couples-controls (parents-child) per patient, matched age, sex assigned at birth, and ethnic origin, (*n =* 126)	Retrospective case study vs. control group
Brinkmann et al. (2007)	Females (*n =* 11)	19–40	SW (*n =* 8) SV (*n =* 3)	46, XX	II–V	Two SW first assigned as boys, reassigned as girls (9 months and 2 years of age)	Patients and medical records	Germany	DSD (5α RD/17βHSD, CAIS, PAIS, GD) (*n =* 26)	Retrospective cohort
Callens et al. (2016)	Females (*n =* 41)	16–46	SW (*n =* 33) SV (*n =* 6) Late onset (*n =* 2)	46, XX	Did not use Prader scores, but they used level of confluence of the vagina and the urethra at birth	Females	Patients	The Netherlands	University students males (*n =* 46) females (*n =* 79)	Not mentioned
Dittmann et al. (1992)	Females (*n =* 34)	11–41	SW (*n =* 12) SV (*n =* 20) Unknown (*n =* 2)	Unknown	Unknown	Unknown	Patients	Germany	Sisters, *n =* 14	Comprehensive interview study
Ehrhardt et al. (1968a)	Females (*n =* 23)	19–55	Late-treated (after eight years of age) (SV 21-OHD)	Unknown	Eighteen had gone through clitorectomy	All had been declared females at birth except one, who was reassigned female at 14 months of age	Patients	USA	No	Cross-sectional
Ehrhardt et al. (1968b)	Females (*n =* 15)	5–16	Early treated (from infancy on)	46, XX	Unknown	Seven assigned males at birth reassigned as females within the first seven months of life	Patients Mothers	USA	Matched control group (*n =* 15) assembled from two public schools	Cross-sectional
Ehrhardt (1979)	Females (*n =* 13)	11–24	Early treated	46, XX	Unknown	Unknown	Patients	USA	No	Cross-sectional
Fagerholm et al. (2011)	Females (*n =* 15)	15–36	SW (*n =* 3) SW (*n =* 12)	46, XX	Unknown	Unknown	Patients	Finland	Age matched controls (*n =* 900)	Not mentioned
Falhammar et al. (2012)^*^	Males (*n =* 32)	19–67	SW 21-OHD (*n =* 17) SW 3β-HSD2D (*n =* 1) SV 21-OHD (*n =* 12) NC 21-OHD (*n =* 2)	46, XY *n =* 31 46, XX *n =* 1	V *n =* 1 VI *n =* 30 Other^#^ *n =* 1	Male	Patients	Sweden	Age matched controls (*n =* 32)	Case-control
Falhammar et al. (2017)	Males (*n =* 221)	15–81	SW (*n =* 83) SV (*n =* 65) NC (*n =* 17)	Unknown	Unknown	Male	Linking of national population-based registers	Sweden	Sex-, age and place of birth matched controls (*n =* 22024)	Population-based national cohort study
Frisen et al. (2009)^**^	Females (*n =* 62)	18–63	SW (*n =* 29) SV (*n =* 27) NC (*n =* 6)	46, XX	I (*n =* 12) II (*n =* 13) III (*n =* 16) IV–V (*n =* 21)	Female	Patients	Sweden	Age-matched (*n =* 62)	Case-control
Gastaud et al. (2007)	Females (*n =* 35)	18–43	21-OHD	Unknown	I (*n =* 4) II (*n =* 6) III (*n =* 11) IV (*n =* 11) V (*n =* 3)	Female	Patients	France	Age- and ethnic-matched controls (*n =* 69)	Cross-sectional
(Gupta et al., 2006)	Females (*n =* 50)	4–26	SW (*n =* 17) SV (*n =* 33)	Unknown	III –IV (*n =* 39)	Female sex assignment at birth (*n =* 39), 9 assigned male sex. Reassigned as females after CAH was diagnosed	Unknown	India	No	Retrospective cohort
Hines et al. (2004)	Females (*n =* 16) Males (*n =* 9)	18–44	21-OHD (*n =* 23) SW (*n =* 22) Unclear (*n =* 2)	Unknown	Unknown	Unknown	Patients	UK	Unaffected female (*n =* 15) and male (*n =* 10) relatives	Cross-sectional
(Johannsen et al., 2006)	Females (*n =* 40)	17–51	CYP21 mutations (*n =* 33) SW (*n =* 21) SV (*n =* 6) NC (*n =* 5) Unclear (*n =* 1) S*tAR* mutations (*n =* 3) CYP17 mutation (*n =* 1) Mutation in the POR gene (*n =* 3)	46, XX	Unknown	Unknown	Patients	Denmark	Civil Registry (*n =* 40)	Case-control study
Jurgensen et al. (2013)	Females (*n =* 74) (grouped with other diagnosis in the category DSD-XX-P-F)	*n =* 30 adolescents (13–16)*n =* 44 adults (17 or older)	Unknown	46, XX	The majority of the participants had surgery	Unknown	Patients and parents	Germany, Austria, and Switzerland	Control data from the secondary school survey in Northern Germany (unpublished) *n =* 546,36 No control data for adults	Observational study
Kanhere et al. (2015)	Females (*n =* 27)	14–26 or older (exact age not available)	21-OHD SW (*n =* 17) SV (*n =* 10)	Unknown	Mildly to moderately virilized genitalia	Unknown	Patients	USA	No	Not mentioned
Khorashad et al. (2017)	Females (*n =* 18)	14–26	Unknown	46, XX	I–V	Females	Patients and parents	Iran	CAIS (*n =* 19)	Not mentioned
Kuhnle et al. (1993)	Females (*n =* 45)	Above 18 years of age	21-OHD SW (*n =* 20) SV (*n =* 17) NC (*n =* 8)	Unknown	I 7.8% II 17.5% III 35.1% IV 36.1% V 1.8%	Females	Patients	Germany	Hospital staff and families (*n =* 46)	Cross-sectional
Lee et al. (2010)	Males (46, XX) (*n =* 12)	35–69	21-OHD	46, XX	IV–V	Ten raised as males, Two raised as females	Patients and physicians	USA	No	Case series
Lesma et al. (2014)	Females (*n =* 12)	24–31	Unknown	Unknown	Unknown	Unknown	Patients	Italy	Healthy university students (*n =* 12)	Case series
Lev-Ran (1974)	Females (*n =* 18)	18 (13)−43	Late-treated (youngest was 11 years)	Unknown	Fourteen had clitorectomy performed	Unknown	Patients	USSR	No	Not mentioned
Liang et al. (2008)	Females (*n =* 11)	8–25	3 SW 8 SV	46, XX	All were born with varying degree of virilization	Females	Parents and patients	Taiwan	No	Cross-sectional
May et al. (1996)	Females (*n =* 19)	18–37	21-OHD	Unknown	II–III (*n =* 5) IV–V (*n =* 13)	Unknown	Patients	UK	Diabetes mellitus (*n =* 17)	Comparative study
Meyer-Bahlburg et al. (2008)	Females (*n =* 143)	18–61	21-OHD SW (*n =* 40) SV (*n =* 21) NC (*n =* 82)	46, XX	Unknown	Unknown	Patients	USA	Sisters and female cousins (*n =* 24)	Cross-sectional
Money et al. (1984)	Females (*n =* 30)	17–26	Unknown	46, XX	All born with clitoromegaly without urethral closure to form a penile meatus and without complete labioscrotal fusion	Females	Patients	USA	AIS (*n =* 15) and MRKH (*n =* 12)	Not mentioned
Morgan et al. (2005)	Females (*n =* 18)	18–36	Unknown	46, XX	Unknown	Females	Patients	UK	No	Cross-sectional
Mulaikal et al. (1987)	Females (*n =* 80)	18–69	40 SW 40 SV All had 21-OHD	46, XX	Unknown	Unknown	Patients	USA	No	Not mentioned
Slijper et al. (1992)	Females (*n =* 18)	16–33	8 SW 1 SV 1 11β-OHD The rest is unknown	46, XX	8 had gender reassignment (6 at 0–3 months, 2 at 6 months)	Unknown	Patients and parents	The Netherlands	Patients with other DSD (*n =* 41)	Not mentioned
Zucker et al. (1996)	Females (*n =* 31)	18–40	19 SW 12 SV	46, XX	Unknown	Unknown	Patients	Canada	Sisters and female cousins (*n =* 15)	Not mentioned

* *Some of the data extracted from another publication (Lolis et al., 2018)*;

** *some of the data extracted from other publications on the same cohort (Hagenfeldt et al., 2008; Nordenskjold et al., 2008)*.

# *Severe penoscrotal hypospadias, micropenis, cryptorchidism, and bifid scrotum were found on the patient with 3βHSD2D. 21-OHD, 21-hydroxylase deficiency; 3βHSD2D, 3β-hydroxysteroid dehydrogenase type 2 deficiency*.

Sexual orientation was measured using a myriad of different constructs and measures across the 30 studies. Examples of different constructs are self-identified sexual orientation, sexual attraction, and sexual relationships (Table 2). In addition, the categorization and reporting of subgroups varied across studies. As an example, some studies reported frequencies of people with heterosexual *and* homosexual orientation in one group (describing variations in sexual orientation across categories), whereas other studies reported frequency as heterosexual or homosexual orientation (binary categorization). In order to be true to the original study, results are reported in the same way as categorized and measured by the authors of the respective studies.

**Table 2:** Overview of included articles studying sexual orientation in people with congenital adrenal hyperplasia with measures and findings.

**References**	**Sample size**	**Measure**	**Findings**
Binet et al. (2016)	*n =* 21 females	Interview (developed for the purpose of the study)	CAH sample: Heterosexual (76%, *n =* 16) Bisexual (14%, *n =* 3) Homosexual (10%, *n =* 2) Control group: Heterosexual (90%, *n =* 57) Bisexual (6%, *n =* 4) Homosexual (3%, *n =* 2)
Brinkmann et al. (2007)	*n =* 11 females	Questionnaire (developed for the purpose of the study)	SW CAH: Heterosexual (50%, *n =* 4) Bisexual (25%, *n =* 2) Homosexual (12%, *n =* 1) SV CAH: Heterosexual (100%, *n =* 3)
Callens et al. (2016)	*n =* 41 females	Adapted version of the gender/sex questionnaire Klein Sexual Orientation Grid	Bisexual or homosexual (16%, *n =* 6/38)
Dittmann et al. (1992)	*n =* 34 females	Semi-structured interview (developed for the purpose of the study)	Total sample: Wish for/current homosexual relationship (20%) 16-20 years: Wish for/current homosexual relationship (26%) Older than 21 years: Wish for/current homosexual relationship (44%)
Ehrhardt et al. (1968a)	*n =* 23 females	Interview questionnaire (developed for the purpose of the study)	Heterosexual experiences and no homosexual experiences (48%, *n =* 11) Heterosexual experiences and occasional homosexual experiences (9%, *n =* 2) Frequent heterosexual and homosexual experiences (9%, *n =* 2) Very limited hetero-/homosexual experiences (4%, *n =* 1) Heterosexual experience: Homosexual dreams (45%, *n =* 5) Bisexual experience: Hetero-/homosexual dreams (13%, *n =* 3)
Ehrhardt et al. (1968b)	*n =* 15 females	Interview (described as standard data schedule of topics)	No evidence of romantic interest in women in any of the sub-groups
Ehrhardt (1979)	*n =* 13 females	Interview (developed for the purpose of the study)	Heterosexual dating interest (62%, *n =* 8) Not very interested (38%, *n =* 5) Active bisexual relationship (8%, *n =* 1) Homosexual crush in fantasy (8%, *n =* 1)
Fagerholm et al. (2011)	*n =* 15 females	FSFI (for participants over 18 years of age)	Heterosexual relationships (86%, *n =* 6) Both genders (14%, *n =* 1)
Falhammar et al. (2012)	*n =* 32 males (46, XY *n =* 31 46, XX *n =* 1)	Questionnaire (developed for the purpose of the study)	Homosexual males: 0% Control group: 3%, *n =* 1/32
Falhammar et al. (2017)	*n =* 221 males	Use of national population-based registry	Registered partnerships males CAH: 0% Control group: 0.05%
Frisen et al. (2009)	*n =* 62 females	Questionnaire (developed for the purpose of the study)	Bi-/homosexual orientation (19%, *n =* 10/53) Control group: (2%, *n =* 1) No answer: CAH (*n =* 9) and controls (*n =* 5) Bi-/homosexual orientation of subgroups: Null genotype (SW): 50% I2 splice mutation (SW): 23% I172N (SV): 5% V281L (NC): 0%
Gastaud et al. (2007)	*n =* 35 females	FSFI Interview (developed for the purpose of the study)	Homosexual inclination (20%, *n =* 7) Control group (6%, *n =* 4/69)
Gupta et al. (2006)	*n =* 50 females (sexual orientation assessed in 19 adolescents and adults)	Questionnaire and interview (developed for the purpose of the study)	**Adolescents** Did not want to talk about sexual orientation (16%, *n =* 3) Wanted a male partner (94%, *n =* 16/17) Wanted a female partner (6%, *n =* 1)
Hines et al. (2004)	*n =* 25 16 females 9 males (Karyotype unknown)	Questionnaire (developed for the purpose of the study)	Recent sexual behavior - Females: Heterosexual (69%, *n =* 11/16) Bisexual (25%, *n =* 4) Homosexual (6%, *n =* 1) Recent sexual behavior - Males: Bi-/homosexual behavior (0%)
Johannsen et al. (2006)	*n =* 33 females	Questionnaire (developed for the purpose of the study) Structured interview (developed for the purpose of the study)	Homosexual relationships (15%, *n =* 5) Controls: Homosexual relationships (100%)
Jurgensen et al. (2013)	*n =* 74 females	UGDS DSD-Questionnaire for Adolescents (developed for the purpose of the study) DSD-Questionnaire for Adults (developed for the purpose of the study)	**Adolescents** Falling in love with: Opposite gender (76%, *n =* 22/29) Both genders (3%, *n =* 1/29) Same gender (3%, *n =* 1/29) Current relationships: Heterosexual (21%, *n =* 3/6) Homosexual (3%, *n =* 1/33). **Adults** Love relationships: Heterosexual (54%, *n =* 25/46) Bisexual (9%, *n =* 4/46) Homosexual (4%, *n =* 2/46) Sexually attracted by: Opposite gender (78%, *n =* 35/46) Both genders (7%, *n =* 3/46) Same gender (11%, *n =* 5/46) Sexual relationships: Heterosexual (62%, *n =* 23/46) Bisexual (16%, *n =* 6/46) Homosexual (5%, *n =* 2/46)
Kanhere et al. (2015)	*n =* 27 females	Questionnaire (developed for the purpose of the study), but derived from generic and validated questionnaires	Only/mostly male sexual partner (62%, *n =* 15/24) Male or female sexual partner (21%, *n* = 5/24) Only/mostly female sexual partner (16%, *n =* 4/24)
Khorashad et al. (2017)	*n =* 18 females	Interview (three items, developed for the purpose of the study)	Heterosexual dreams (44%, *n =* 8) Exclusively Homosexual experiences (11%, *n =* 2) Homosexual fantasies (17%, *n =* 3) Homosexual dreams (44%, *n =* 8)
Kuhnle et al. (1993)	*n =* 45 females	BIQ ASQ	Homosexual CAH (4%, *n =* 2/45) Control group (2%, *n =* 1/46)
Lee et al. (2010)	*n =* 12 males (all 46, XX)	Information from patients' physician	Heterosexual orientation (100%, *n =* 12), both in behavior and fantasies
Lesma et al. (2014)	*n =* 12 females	Semi-structured interview (developed for the purpose of the study) FSDS FSFI	Stable heterosexual relationships (92%, *n =* 11) Stable homosexual relationships (8%, *n =* 1)
Lev-Ran (1974)	*n =* 18 females	Simple questioning	All were heterosexual (100%)
Liang et al. (2008)	*n =* 11 females	CHQ RSTBQ Semi -structured Gender Identity Interview	Bisexual (9%, *n =* 1) Homosexual (9%, *n =* 1)
May et al. (1996)	*n =* 19 females	Interview (developed for the purpose of the study)	Homosexual relationships (11%, *n =* 2) Sexual appreciation of females (16%, *n =* 3)
Meyer-Bahlburg et al. (2008)	*n =* 143 females	Interview with a clinical psychologist (SEBAS-A) For each sexual orientation variable, interviewers' ratings were based on the Kinsey Rating Scale.	SW-CAH: Heterosexual (84%, *n =* 32/38) Homosexual (16%, *n =* 6/38) SV-CAH: Heterosexual (95%, *n =* 20/21) Homosexual (5%, *n =* 1/21) NC-CAH: Heterosexual (89%, *n =* 71/80) Homosexual (11%, *n =* 9/80)
Money et al. (1984)	*n =* 30 females	Interview (developed for the purpose of the study)	Heterosexual only (40%, *n =* 12) Bisexual (20%, *n =* 6) Homosexual only/predominantly (17%, *n =* 5) History of erotosexual status: Heterosexual (40%, *n =* 12) Bisexual (37%, *n =* 11)
Morgan et al. (2005)	*n =* 18 females	Structured Clinical Interview for DSM-IV-R GHQ-30 SAS-modified	Bisexual (11%, *n =* 2) Homosexual (11%, *n =* 2)
Mulaikal et al. (1987)	*n =* 80 females	Questionnaire (developed for the purpose of the study)	Total sample: Bi-/homosexual (5%, *n* = 4) No sexual experience (38%, *n =* 30) SW- CAH: Heterosexual (53%, *n =* 21/40) Bi-/homosexual (3%, *n =* 1/40) Unknown (45%, *n =* 18/40) SV-CAH: Heterosexual (63%, *n =* 24/40) Bi-/homosexual (8%, *n =* 3/40) Unknown (30%, *n =* 12/40)
Slijper et al. (1992)	*n =* 18 females	Standardized interview	All heterosexual (100%)
Zucker et al. (1996)	*n =* 31 females	EROS Interview (developed for the purpose of the study) Kinsey-ratings HES-modified	Twelve month global rating: No sexual experiences (27%, *n =* 8) Exclusively heterosexual experiences (73%, *n =* 22) Controls: 100% heterosexual experiences Lifetime global rating: No sexual fantasies (7%, *n =* 2) Exclusively heterosexual fantasies (70%, *n =* 21) Bisexual fantasies (23%, *n =* 7) Controls: 100% heterosexual experiences

*FSFI, Female Sexual Function Index; UGDS, Utrecht Gender Dysphoria Scale for adolescents; BIQ, The Body Image Questionnaire; ASQ, Attitudes toward Sexuality Questionnaire; FSDS, Female Sexual Distress Scale; CHQ, Chinese Health Questionnaire; RSTBQ, Recalled Sex-Typed Behavior Questionnaire; SEBAS-A, Sexual Behavior Assessment Schedule; Kinsey; GHQ, General Health Questionnaire 30; SAS, Social Adjustment Scale, modified; HES, Heterosexual Experience Scale, modified (Zuckerman, 1973, 1988)*.

#### Sexual Orientation (46, XX Assigned Females)

##### Sexual identity

A majority of assigned females with CAH *self-identified* (defined themselves) as heterosexual (Money et al., 1984; Mulaikal et al., 1987; Slijper et al., 1992; Kuhnle et al., 1995; Zucker et al., 1996; Morgan et al., 2005; Brinkmann et al., 2007; Liang et al., 2008; Meyer-Bahlburg et al., 2008; Frisen et al., 2009; Falhammar et al., 2012; Jurgensen et al., 2013; Lesma et al., 2014; Binet et al., 2016; Callens et al., 2016), but figures varied widely across studies (40–100%). Among these 15 studies (Money et al., 1984; Mulaikal et al., 1987; Slijper et al., 1992; Kuhnle et al., 1995; Zucker et al., 1996; Morgan et al., 2005; Brinkmann et al., 2007; Liang et al., 2008; Meyer-Bahlburg et al., 2008; Frisen et al., 2009; Falhammar et al., 2012; Jurgensen et al., 2013; Lesma et al., 2014; Binet et al., 2016; Callens et al., 2016), five were conducted more than 20 years ago (two in the eighties, three in the nineties). Five of the studies had samples with less than 20 individuals, and one study had a sample of 21 individuals. Results indicate that fewer assigned females reported homosexual (3–20%) or bisexual orientation (3.4–37%). One study (Frisen et al., 2009) reported that 19% of females within a sample of 62 females were non-heterosexual. Another article (Ehrhardt et al., 1968a) summarizes results in three different categories of sexual orientation: heterosexual with no homosexual experience (48%), heterosexual with occasional homosexual experience (9%), and frequent heterosexual and homosexual experiences (9%). Another study reported that 16% were homosexual or bisexual, without differentiating between these two categories (Callens et al., 2016).

##### Sexual identity in subgroups of CAH

Three studies investigated sexual orientation in subgroups of CAH (Mulaikal et al., 1987; Brinkmann et al., 2007; Frisen et al., 2009). One of these studies was published in 1987 (Mulaikal et al., 1987), while another (Brinkmann et al., 2007) included 11 individuals who were categorized into smaller subgroups. Mulaikal et al. (1987) found a higher frequency of homosexual orientation in females with SV CAH (8%), compared with 3% in the SW group. Brinkmann et al. (2007) reported that all females with SV CAH were heterosexual, while 50% of the females with SW CAH were heterosexual, 25% bisexual, and 12% homosexual. Using CAH genotypes, Frisen et al. (2009) related sexual orientation in females with CAH to severity. A total of 50% of individuals with the most severe genotype (null mutations) indicated that they were bisexual or homosexual, whereas the figure was 30% for those with the second-most severe genotype (I2 splice mutation). These two mutations correspond more or less to the SW form. For females with the I172N mutation (associated with the SV form), 5% reported that they were non-heterosexual, whereas all females with the least severe mutation, V281L (associated with the NC-form), reported heterosexuality.

##### Sexual inclination and orientation

Three studies investigated sexual inclination (Dittmann et al., 1992; Gastaud et al., 2007; Meyer-Bahlburg et al., 2008), which was defined as the measurement of erotic homosexual dreams and fantasies, active homosexual experience, and sexual identity (Gastaud et al., 2007). One of these studies was published almost three decades ago (Dittmann et al., 1992). Gastaud et al. (2007) found that 20% of his sample of 35 females reported homosexual inclinations. Dittmann et al. (1992) explored sexual orientation, described as whether participants wished for and/or actually were in homosexual relationships, and found that this was the case for 20% in a sample of 34 females with CAH. For participants over 16 years of age, 26% reported that they wished for and/or were in a homosexual relationship, while this figure had increased to 44% for participants above 21 years of age (Dittmann et al., 1992).

##### Sexual inclination and orientation in subgroups of CAH

Homosexual inclination was expressed by 43% of the females with Prader IV and V (Gastaud et al., 2007). Meyer-Bahlburg et al. (2008) found a significant increase in bisexual/homosexual orientation expressed through imagery in females with classic CAH (SV and SW subgroups combined) compared with non-CAH controls (33–47 vs. 5%). Females with SW CAH were found to have a higher frequency of bisexual/homosexual orientation than females with SV CAH and females with NC-CAH (47 vs. 33 vs. 11%; Meyer-Bahlburg et al., 2008).

##### Sexual attraction

Three studies used the terminology sexual attraction, including falling in love with or feeling sexual attraction and appreciation for another person (Ehrhardt, 1979; May et al., 1996; Jurgensen et al., 2013). One of the studies was more than 25 years old (May et al., 1996), while another was published more than 40 years ago (Ehrhardt, 1979). May et al. (1996) reported a strong sense of sexual appreciation of females in 16% of their sample of 19 females in their study published in the nineties. In a more than 40 year old study, Ehrhardt (1979) explored female participants' interest in dating boys and found that 62% of females in a sample of 13 were interested, while 38% were less interested. Jurgensen et al. (2013) included both adolescents and adults and found that 76% of their adolescents sample fell in love with the opposite gender, 3% with both genders, and 3% with the same gender. In the adult sample, 78% felt sexual attraction to the opposite gender, 11% to the same gender, and 7% to both genders.

##### Sexual fantasies and sexual dreams

Six studies explored sexual fantasies and dreams in individuals with CAH (Ehrhardt et al., 1968a; Lev-Ran, 1974; Ehrhardt, 1979; Zucker et al., 1996; Lee et al., 2010; Khorashad et al., 2017). Two of the studies (Lev-Ran, 1974; Khorashad et al., 2017) were conducted in countries were homosexuality may not be well-accepted (Iran and USSR), which might have affected results. Five of the six studies were published more than 25 years ago, three of them in the sixties and seventies (Ehrhardt et al., 1968a; Lev-Ran, 1974; Ehrhardt, 1979). In addition, all six studies had low samples sizes, ranging from 12 to 31. Results showed that among 23 females with heterosexual experiences (Ehrhardt et al., 1968a), 46% had homosexual dreams, and among those with bisexual experience, 13% had heterosexual/homosexual dreams. Among 13 females with CAH (Ehrhardt, 1979), 8% reported a crush in fantasy on another girl. Another study found that 17% of 18 included females had fantasies involving other females and 44% had sexual dreams including females (Khorashad et al., 2017). In another study (*n* = 31), 23% had bisexual fantasies and 70% had exclusively heterosexual fantasies (Zucker et al., 1996). Lev-Ran (1974), who investigated homosexuality in USSR in the seventies, found that females with CAH reported 100% heterosexual erotic dreams.

##### Erotosexual arousal (imagery/and or activity)

Two studies (Money et al., 1984; Callens et al., 2016) measured sexual orientation by investigating imagery and behavior (actual sex partner experiences and activity). One of these studies was conducted almost 40 years ago (Money et al., 1984), while the second and most recent one (Callens et al., 2016), unfortunately includes pixelated figures that are blurry and indistinct both on the online and printed versions of the article. The other study (Money et al., 1984) found that 40% of females with CAH had a history of heterosexual imagery and/or activities, while 37% had a history of bisexual imagery and/or activities.

##### Sexual behavior/experiences/relations/practices/activity

A total of nine studies have explored sexual orientation by measuring sexual behaviors, experiences, relations (potentially different to relationships), practices, and/or sexual activities (Ehrhardt et al., 1968a; Lev-Ran, 1974; Zucker et al., 1996; Hines et al., 2004; Gastaud et al., 2007; Meyer-Bahlburg et al., 2008; Lee et al., 2010; Callens et al., 2016; Khorashad et al., 2017). Five of the studies had small samples, ranging from 16 to 23 (Ehrhardt et al., 1968a; Lev-Ran, 1974; Hines et al., 2004; Lee et al., 2010; Khorashad et al., 2017) and three of the studies were more than 25 or 50 years old (Ehrhardt et al., 1968a; Lev-Ran, 1974; Zucker et al., 1996). One study measured recent sexual behavior in 16 females with CAH and found that 69% reported heterosexual behavior, 25% bisexual behavior, and 6% homosexual behavior (Hines et al., 2004). Ehrhardt et al. (1968a), a study published in the sixties, and found that 9% of late-treated females with CAH had frequent heterosexual and occasional homosexual experiences, while 9% had frequent heterosexual and frequent homosexual experiences. In a study from the USSR and published in 1974 (Lev-Ran, 1974), no females in a sample of 18 reported homosexual experiences, whereas 73% classified themselves as having had exclusively heterosexual behaviors for the last 12 months in a 25 year old study (Lev-Ran, 1974; Zucker et al., 1996). When asked about lifetime behaviors, 3% reported bisexual experiences, while 80% classified their experiences as exclusively heterosexual (Zucker et al., 1996). Sexual behavior was also investigated in Callens et al. (2016), but as mentioned above, figures were difficult to read. Khorashad et al. (2017) used the concept of sexual contact to measure sexual orientation and found that 17% of the females reported sexual contact with men, while 12% had experienced sexual contact with females.

##### Sexual behavior/experiences/relations/practices/activity in subgroups of CAH

Meyer-Bahlburg et al. (2008) found a higher number of homosexual experiences (genital sex) in females with SW CAH (15%) compared with non-CAH controls (0%). A total of 5% of the females with SV CAH and 11% of the females with NC-CAH had homosexual experiences (Meyer-Bahlburg et al., 2008). Gastaud et al. (2007) also explored subgroups of CAH and reported that almost 6% of females with Prader IV and V stated that they were homosexual or had active homosexual experiences, compared with 2% in a national (French) population sample.

##### Sexual relationships

Ten studies investigated sexual orientation by asking about sexual relationships (Ehrhardt, 1979; Zucker et al., 1996; Johannsen et al., 2006; Meyer-Bahlburg et al., 2008; Lee et al., 2010; Fagerholm et al., 2011; Jurgensen et al., 2013; Lesma et al., 2014; Callens et al., 2016; Khorashad et al., 2017). Five of these studies had <20 individuals in their samples (Ehrhardt, 1979; Lee et al., 2010; Fagerholm et al., 2011; Lesma et al., 2014; Khorashad et al., 2017), and two of the studies were more than 20 years old (Ehrhardt, 1979; Zucker et al., 1996). Johannsen et al. (2006) found that 15% of females with CAH in a sample 33 were in homosexual relationships. Another study with a sample of 74 females found that 62% were engaged in heterosexual relationships, 5% in homosexual relationships, and 16% in bisexual relationships (Jurgensen et al., 2013). Another study reported the frequency of experienced homosexual relationships to be 11% in a sample of 19 females (May et al., 1996), while another study measured the frequency of exclusively homosexual experiences and also found it to be 11% (Khorashad et al., 2017). Yet another study reported 86% of heterosexual relationships in 15 females, while the remaining 14% had sexual relationships with both male and female partners (Fagerholm et al., 2011). In 29 adolescent females, 21% reported being in current heterosexual relationships, while 3% had previous homosexual relationships (Jurgensen et al., 2013). In a sample of 46 adult females, 54% were in heterosexual current love and sexual relationships, 4% in homosexual, and 9% were in bisexual current love and sexual relationships (Jurgensen et al., 2013). Ehrhardt (1979) found that 1 of 13 females (8%) with CAH reported active bisexual love relationships, a study that was published in 1979. Another study, with only 12 participants, 92% reported a stable satisfactory heterosexual relationship, while 8% reported a homosexual relationship (Lesma et al., 2014).

##### Sexual relationships in subgroups of CAH

Meyer-Bahlburg et al. (2008) found a higher number of females with SW CAH (21%) in homosexual relationships compared with non-CAH controls (0%). A total of 5% of females with SV CAH and 4% of females with NC CAH were in homosexual relationships (Meyer-Bahlburg et al., 2008).

##### Preferred sexual partner

One study used the terminology preferred sexual partner to measure sexual orientation (Kanhere et al., 2015). This study included 27 females with CAH and reported that 62% preferred male partners, 21% either male or female partners, and 16% only or mostly female partners (Kanhere et al., 2015).

##### Love relationships

Two studies measured love relationships as a way to capture sexual orientation (Dittmann et al., 1992; Jurgensen et al., 2013). Jurgensen et al. (2013) included both adolescents and adults in their sample. Among the adolescents, 76% reported that they did not have a current love relationship, 21% were in current love relationships with the opposite gender, and 3% were in current love relationships with the same gender. Among the adults, 33% were not in a current love relationship, 54% were in a love relationship with the opposite gender, 9% were in relationships with males and females, and 4% were in a love relationship with the same gender.

##### Love relationship in subgroups of CAH

Dittmann et al. (1992) found that 17% of females over 16 years of age with SW CAH and 18% with SV CAH wanted or had a long term and steady relationship with a partner of the same sex. For those older than 21, 40% of females with SV CAH wanted or had a long term or steady relationship with females, compared to none of the females with SW CAH.

##### Preferred life partner and romantic interest

Two studies investigated adolescents' imagined future romantic interest (Ehrhardt et al., 1968b; Gupta et al., 2006). Gupta et al. (2006) explored unmarried female adolescents' preferred life partner in India. A total of 94% wanted to marry a male partner, while 6% wanted to marry a female. The second study was published more than 50 years ago (Ehrhardt et al., 1968b), and studied early-treated young females with CAH, aged 5–16 years. They found no evidence of romantic interest in females, neither in the control group nor in those with CAH. The results also showed that only 9 of the 15 females (60%) fantasized about wedding and marriage, compared to all females in the control group.

#### Sexual Orientation (46, XY Males)

##### Sexual behaviors and partnerships

Three studies investigated sexual orientation in males 46, XY (Hines et al., 2004; Falhammar et al., 2012, 2017). The number of registered partnerships, which could indicate homosexual orientation, was explored in Falhammar et al. (2017). None of the 221 males with CAH reported being in a registered partnership, compared to 0.05% of 22,024 matched controls. In another study from the same research group, none of the 32 males with CAH (46, XY *n* = 31, 46, XX *n* = 1) expressed any homosexuality compared to 3% of the 32 matched controls (Falhammar et al., 2012). Similar figures were confirmed in a third study, where none of the nine males reported current bisexual or homosexual behavior (Hines et al., 2004).

#### Sexual Orientation (46, XX Males)

##### Life partner, sexual behaviors, and fantasies

Two studies (Lee et al., 2010; Falhammar et al., 2012) investigated assigned males with CAH (46, XX). The first study, based on a sample of 12 males with CAH (all 46, XX), measured sexual behaviors and fantasies, indicating a 100% sexual orientation to females (Lee et al., 2010). The second study of males with CAH included one male individual with 46, XX who expressed sexual orientation to females only, was married to a woman, and had two adopted children (Falhammar et al., 2012).

#### Summary of the Results

To simplify the results, Tables 3 and 4 present a summary of all included studies.

**Table 3:** Summary of sexual orientation in people with congenital adrenal hyperplasia.

**Concept**	**Sex**	**Studies in chronological order**	**Publication year range**	**Age range (years)**	**Findings, Ranges in percent**
Sexual identity	Females	Money et al. (1984); Mulaikal et al. (1987); Slijper et al. (1992); Kuhnle et al. (1995); Zucker et al. (1996); Morgan et al. (2005); Brinkmann et al. (2007); Liang et al. (2008); Meyer-Bahlburg et al. (2008); Frisen et al. (2009); Falhammar et al. (2012); Jurgensen et al. (2013); Lesma et al. (2014); Binet et al. (2016); Callens et al. (2016).	1984–2016	8–69	Heterosexual: 40–100 Bisexual: 3,4–37 Homosexual: 3–20 Bi-/homosexual:0–50
Sexual inclination and orientation	Females	Dittmann et al. (1992); Gastaud et al. (2007); Meyer-Bahlburg et al. (2008).	1992–2008	11–61	Homosexual: 20–44 Bi-/homosexual inclination: 33–47
Sexual attraction	Females	Ehrhardt (1979); May et al. (1996); Jurgensen et al. (2013)	1979–2013	11–37	Heterosexual: 62–78 Bisexual: 3–7 Homosexual: 3–16
Sexual fantasies and dreams	Females	Ehrhardt et al. (1968a); Lev-Ran (1974); Ehrhardt (1979); Zucker et al. (1996); Lee et al. (2010); Khorashad et al. (2017).	1968–2017	11–69	Heterosexual: 70–100 Bisexual fantasies: 23 Homosexual: 8–46 Homo-/bisexual: 13
Erotosexual arousal (imagery/and or activity)	Females	Money et al. (1984); Callens et al. (2016).	1984–2016	16–46	Heterosexual: 40 Bisexual: 37
Sexual behavior/ experiences/relations/ practices/activity	Females	Ehrhardt et al. (1968a); Lev-Ran (1974); Zucker et al. (1996); Hines et al. (2004); Gastaud et al. (2007); Meyer-Bahlburg et al. (2008); Lee et al. (2010); Callens et al. (2016); Khorashad et al. (2017).	1968–2017	14–69	Heterosexual: 17–100 Bisexual: 3–25 Homosexual: 0–16
Sexual relationships	Females	Ehrhardt (1979), Zucker et al. (1996), Johannsen et al. (2006), Meyer-Bahlburg et al. (2008), Lee et al. (2010), Fagerholm et al. (2011), Jurgensen et al. (2013), Lesma et al. (2014), Callens et al. (2016), Khorashad et al. (2017).	1979–2017	11–69	Heterosexual: 54–92 Bisexual: 8–16 Homosexual: 3–15
Preferred sexual partner	Females	Kanhere et al. (2015)	2015	14–26	Heterosexual: 62 Bisexual: 21 Homosexual: 16
Love relationships	Females	Dittmann et al. (1992), Jurgensen et al. (2013).	1992–2013	11–41	Heterosexual: 21–54 Bisexual: 9 Homosexual:3–40
Preferred life partner and romantic interest	Females	Ehrhardt et al. (1968b), Gupta et al. (2006).	1968–2006	4–26	Heterosexual: 94 Homosexual: 0-6
Sexual behaviors and partnerships	Males (46, XY)	Hines et al. (2004), Falhammar et al. (2012), Falhammar et al. (2017).	2004–2017	15–81	Homosexual: 0
Life partner, sexual behaviors, and fantasies	Males (46, XX)	Lee et al. (2010), Falhammar et al. (2012).	2010–2012	35–69	Homosexual: 0 Bisexual: 0

**Table 4:** Definition of constructs of sexual orientation as described in the results by the authors in this systematic review.

**Construct used**	**Definition**	**Studies in chronological order**
Sexual identity	The individual's self-conception of being homosexual, bisexual, and/or heterosexual.	Money et al., 1984; Mulaikal et al., 1987; Slijper et al., 1992; Kuhnle et al., 1995; Zucker et al., 1996; Morgan et al., 2005; Brinkmann et al., 2007; Liang et al., 2008; Meyer-Bahlburg et al., 2008; Frisen et al., 2009; Falhammar et al., 2012; Jurgensen et al., 2013; Lesma et al., 2014; Binet et al., 2016; Callens et al., 2016
Sexual inclination and orientation	Erotic sexual dreams and fantasies, active sexual experience, and sexual identity.	Dittmann et al., 1992; Gastaud et al., 2007; Meyer-Bahlburg et al., 2008.
Sexual attraction	Fell in love with/sexual attraction/sexual appreciation.	Ehrhardt, 1979; May et al., 1996; Jurgensen et al., 2013.
Sexual fantasies and dreams	Sexual fantasies, including crush in fantasies, and sexual dreams.	Ehrhardt et al., 1968a; Lev-Ran, 1974; Ehrhardt, 1979; Zucker et al., 1996; Lee et al., 2010; Khorashad et al., 2017.
Erotosexual arousal	Imagery/and or activity.	Money et al., 1984; Callens et al., 2016.
Sexual behavior/experiences/relations/practices/activity	Recent sexual behavior; frequent heterosexual and occasional homosexual experience; frequent heterosexual experience and frequent homosexual experience; heterosexual/homosexual in behavior.	Ehrhardt et al., 1968a; Lev-Ran, 1974; Zucker et al., 1996; Hines et al., 2004; Gastaud et al., 2007; Meyer-Bahlburg et al., 2008; Lee et al., 2010; Callens et al., 2016; Khorashad et al., 2017.
Sexual relationships	Heterosexual/bisexual/homosexual sexual relationships.	Ehrhardt, 1979; Zucker et al., 1996; Johannsen et al., 2006; Meyer-Bahlburg et al., 2008; Lee et al., 2010; Fagerholm et al., 2011; Jurgensen et al., 2013; Lesma et al., 2014; Callens et al., 2016; Khorashad et al., 2017.
Preferred sexual partner	Preferred gender of sexual partner	Kanhere et al., 2015.
Love relationships	Current love relationship	Dittmann et al., 1992; Jurgensen et al., 2013.
Preferred life partner and romantic interest	Imagined future romantic interest; fantasizing about wedding and marriage	Ehrhardt et al., 1968b; Gupta et al., 2006
Sexual behaviors and partnerships	Registered partnerships	Hines et al., 2004; Falhammar et al., 2012, 2017.
Life partner, sexual behavior, and fantasies	My sexual behavior has been with… My sexual fantasies have been with…	Lee et al., 2010; Falhammar et al., 2012.

### Outcome Measures

An overview of all outcome measures can be found in Table 2. Among the 30 included studies, 19 (63%) used a self-designed questionnaire and/or a clinical/research-oriented interview (Ehrhardt et al., 1968a; Lev-Ran, 1974; Ehrhardt, 1979; Money et al., 1984; Ellis and Ames, 1987; Mulaikal et al., 1987; Dittmann et al., 1992; May et al., 1996; Hines et al., 2004; Morgan et al., 2005; Gupta et al., 2006; Johannsen et al., 2006; Brinkmann et al., 2007; Frisen et al., 2009; Falhammar et al., 2012; Lesma et al., 2014; Binet et al., 2016; Khorashad et al., 2017). One of these studies Kanhere et al. (2015), developed a questionnaire based on validated generic instruments, such as the Body Esteem Scale (Franzoi and Shields, 1984), FSFI (Rosen et al., 2000), Recalled Childhood Gender Identity Scale (Meyer-Bahlburg et al., 2006), and SF-36 (Ware and Sherbourne, 1992; Patel et al., 2007). However, by doing so, the new questionnaire cannot be considered validated. Nine studies (31%) used validated measures such as the Female Sexual Function Index (FSFI) (Gastaud et al., 2007; Fagerholm et al., 2011; Lesma et al., 2014), Utrecht Gender Dysphoria Scale for adolescents (UGDS) (Jurgensen et al., 2013), Body Image Questionnaire (BIQ), Attitudes toward Sexuality Questionnaire (ASQ) (Kuhnle et al., 1995), Chinese Health Questionnaire (CHQ) (Liang et al., 2008), Sexual Behavior Assessment Schedule (SEBAS-A), Kinsey rating scale (Meyer-Bahlburg et al., 2008), EROS, and Kinsey (Zucker et al., 1996), or a standardized but unnamed questionnaire (Slijper et al., 1992). One study used a national population-based registry and used registered partnership as an indication of sexual orientation in males with CAH (Falhammar et al., 2017). Another study collected data from the patient's practitioner's evaluations (Lee et al., 2010). Very few studies had used the same validated measure as other studies: three studies had used the FSFI (Gastaud et al., 2007; Fagerholm et al., 2011; Lesma et al., 2014), and two studies (Zucker et al., 1996; Meyer-Bahlburg et al., 2008) had used the Kinsey scale.

## Discussion

The present systematic review investigated sexual orientation in females and males with CAH with the aim of summarizing the current literature on this topic. No time restriction was imposed on publications, and 11 studies (38%) were conducted more than 20 years ago. This review included a sample of 1201 people with CAH, 274 assigned males at birth (both 46, XY and 46, XX) and 927 assigned females at birth. Twelve of the studies including control groups (Money et al., 1984; Dittmann et al., 1992; Kuhnle et al., 1993; Zucker et al., 1996; Hines et al., 2004; Johannsen et al., 2006; Meyer-Bahlburg et al., 2008; Frisen et al., 2009; Fagerholm et al., 2011; Lesma et al., 2014; Binet et al., 2016; Khorashad et al., 2017) found that rates of non-heterosexual orientation were higher in assigned females with CAH than in controls, whereas no individuals with CAH assigned male (46, XY or 46, XX) expressed any non-heterosexual orientation. The second aim was to provide an overview of the measures used within this field of research, which showed a wide diversity in outcome measures and a preference for unvalidated questionnaires and self-constructed interviews.

We wanted to include studies that assessed sexual orientation in both males and females with CAH, as this has not been done previously. A recent review by Gondim et al. (2018) investigated sexual orientation in assigned females with CAH. While this review constitutes an important contribution to the research field, several differences between Gondim et al.'s review and the present one are worth noting. First, our review included studies on both males and females. Second, we also explored which dimensions of sexual orientation that had been measured in the different studies, widening the scope of the review. Third, Gondim et al. (2018) described their review as descriptive, and limited their search to the PubMed database, using fewer search terms, including only studies published between 1985 and 2016, and included studies with <10 participants. Gondim et al. (2018) included a total of nine studies in their review, in contrast to the present review's inclusion of 30 studies. We therefore believe the current review constitutes a more comprehensive overview of sexual orientation in individuals with CAH.

### Sexual Orientation in Males With CAH

In the present review, three studies included males only (Lee et al., 2010; Falhammar et al., 2012, 2017), and one study included both males and females (Hines et al., 2004). One of the studies including males involved only 46, XX CAH raised as males (Lee et al., 2010). None of the studies had male participants identifying themselves as homosexual or bisexual.

### Sexual Orientation in Females With CAH

Despite the wide variation in outcome measures, categorizations, and concepts used, the conclusion of the present review seems to be that females with CAH have greater likelihood to have a non-heterosexual orientation than females from the general population. Several potential interpretations may help explain these results.

The brain organization theory holds that steroid hormones during fetal development permanently organize the brain for gender, including patterns of sexuality, and is the leading biological theory of sexual orientation in humans (Jordan-Young, 2012; Roselli, 2018). This theory claims that prenatal exposure to sex hormones, such as testosterone, not only triggers the formation of sexual organs, but also impacts the brain throughout one's lifetime, permanently but differently “hardwiring” the brains of males and females (Jordan-Young, 2012). Studies of individuals with CAH may shed light on the brain organization theory, and advocates of this theory will attribute atypical psychosexuality in this group to elevated prenatal androgen levels that have masculinized the brain. Prenatal elevated androgen levels have also been shown to influence children's sex-typical play behaviors (Dittmann et al., 1990; Berenbaum and Hines, 1992; Hall et al., 2004; Hines et al., 2004; Hines, 2006), which may explain why females with CAH have been shown to demonstrate more male-typical rough and “tomboyish” behavior than controls. Evidence exists to link childhood play interests and adult sex orientation (Saghir, 1973; Bell and Hammersmith, 1981; Grellert et al., 1982; Harry, 1982; Bailey and Zucker, 1995). Hence, early hormonal influences affecting sexual differentiation of the brain may permanently influence later behavior, suggesting that sexual orientation is programmed into our brain structures prenatally (Savic et al., 2010).

However, the assumption of a female and a male brain organization that would explain behavioral differences between the two sexes, has also been challenged (Fisher et al., 2018). Effects on prenatal testosterone cannot be the only factor involved in explaining sexual orientation, since not all individuals with CAH are homosexual. Nevertheless, sexual differentiation of the genitals takes place during the first 2 months of pregnancy, while the brain's sexual differentiation starts in the pregnancy's last 4–5 months. Hence, these two processes could be influenced independently, and the degree of masculinization of the genitals may therefore not reflect the degree of masculinization of the brain (Swaab and Garcia-Falgueras, 2009). Further, the influence of prenatal androgen exposure differs from child to child, leading to varying degrees of genital virilisation (ranging from severe to mild). Androgen levels affecting the masculinization of the brain may also vary, leading to individual differences.

Another DSD condition (46, XY), 5 alpha reductase type 2 deficiency (5α-RD-2) may illustrate how the degree of external virilization of the genitalia does not constitute an accurate estimate of brain virilization. During fetal development, the enzyme 5α-reductase-2 is required for the conversion of testosterone into dihydrotestosterone which, in turn, is responsible for the development of the external male genitals (Domenice et al., 2000). Individuals with 5α-RD-2 are often raised as females. However, in this condition, the brain is prenatally exposed to testosterone levels that are normal for males, and many affected individuals, even when raised as females, develop a male gender identity and eventually experience gender incongruence later in life. However, even if they opt for gender affirming surgery frequently, it does not apply to all 5α-RD-2 affected individuals. Hence, the degree of external genital masculinization at birth does not seem to be related to gender incongruence or dysphoria in a systematic way (Cohen-Kettenis, 2005).

Researchers have suggested that several other factors also need to be taken into account when discussing behavioral effects of prenatal androgens on females with CAH (Jordan-Young, 2012; Fisher et al., 2018). First, the masculinization of females with CAH's genitalia may elicit social responses, especially from parents that might explain behavioral changes in the child (Quadagno et al., 1977). Second, behavioral changes in females with CAH may reflect effects of increased androgens also postnatally. Third, CAH is a medical condition, and behavioral changes may reflect the consequences of living with a chronic disease. Fourth, females with CAH have been shown to have impaired clitoral sensation as a result of surgery and ~30–60% do not have a vaginal introitus large or flexible enough to permit heterosexual intercourse (Gastaud et al., 2007; Crouch et al., 2008; Nordenskjold et al., 2008; Balthazart, 2011; Jordan-Young, 2012; Hemesath et al., 2019), and/or heterosexual intercourse may be experienced as painful (Gastaud et al., 2007). Challenging sexual experiences has been suggested to potentially affect preferences toward sexual orientation. Studies have also shed light on how a potential partner's negative reactions to an atypical genital appearance could lead to distress, embarrassment, and/or shame, which could further impact these females' sexual self-perceptions and potentially their sexual interest (Meyer-Bahlburg et al., 2018). From a psychological perspective, one can also ask whether repeated genital examinations and treatment could affect sexuality. Hines (2011b) therefore proposed that the females' negative experiences with heterosexual activities could induce a general aversion toward sexual activity. Physical, medical and psychological factors could therefore be hypothesized to decrease heterosexual interest and lead to homosexual interest (Hines, 2011b). Hence, social learning and experiences, in addition to biological factors, need to be taken into account when exploring the impact of androgens on brain and gender development (Jordan-Young, 2012; Del Giudice, 2019).

Sexual orientation is not necessarily a uniformly immutable trait (Diamond and Rosky, 2016). Researchers have argued that female sexual orientation may be more flexible, fluid, and non-exclusive than male sexual orientation (Diamond, 2007; Bailey, 2009), and possibly also more influenced by cultural and social factors (Baumeister, 2000). Sexual fluidity may therefore partly explain mixed-sex (non-exclusive) attractions, or changes in sexual attraction over time (Savin-Williams et al., 2012). The “coming-out process” of homosexual and bisexual females should also be taken into account, since it has been suggested to be particularly variable among females (Savin-Williams and Diamond, 2000). However, studies in the current review that included control groups still reported higher frequencies of homosexual or bisexual orientation in females with CAH.

Severity of CAH might have influenced the outcomes of sexual orientation in some of the studies included in this review. Meyer-Bahlburg et al. (2008) assessed sexual orientation in females with different phenotypes, and compared them to a control group of unaffected sisters or cousins. The study showed an increased non-heterosexual orientation in all females with CAH, including NC-CAH. This was surprising, since it is assumed that fetuses with NC-CAH are exposed to mild androgen excess that are insufficient to affect sexual differentiation of the genitals. Still, the androgen excess seems to be sufficient to slightly affect the differentiation of the brain (Meyer-Bahlburg et al., 2008), supporting a possible differentiation between masculinization of the genitals and masculinization of the brain. An alternative explanation could be postnatal effects of mild, but continuous androgen excess, affecting brain and gender related behaviors. In line with these results, Dittmann et al. (1992) found that a higher frequency of females with SV CAH wanted or had long-term and/or steady homosexual relationships than females with SW CAH. The authors stated that the females with SW CAH had received earlier and better treatment over their lifetime than the ones with SV CAH, and therefore had been less affected by postnatal androgenization than females with SV CAH. However, and in contrast, Frisen et al. (2009) found the prevalence of non-heterosexual orientation to be higher among females with the more severe geno- and phenotypes. Two other studies (Mulaikal et al., 1987; Slijper et al., 1992), both published more than 25 years ago, suggested that non-heterosexual orientation should rather be explained by physical characteristics of females with CAH's genitals, such as vaginal condition and function, and fear of rejection by male partners, rather than levels of prenatal androgens. Future research is still needed to disentangle the myriad of factors potentially affecting sexual orientation in CAH.

### What Is Sexual Orientation and How Do We Measure It?

In Western cultures, openness to sexual orientation has dramatically changed during the last decades, and homosexual and bisexual individuals' political and civic rights have dramatically improved (Bailey et al., 2016). In the present review, one third of the studies were more than 20 years old, which may have affected participants' willingness to be open about potential bisexual or homosexual orientation. Further, non-heterosexual behavior still remains illegal and is severely punished in much of Africa, Middle East, Caribbean, Oceania, and parts of Asia (Carroll and Itaborahy, 2015; Bailey et al., 2016). Three studies (Lev-Ran, 1974; Gupta et al., 2006; Khorashad et al., 2017) were conducted in countries where sexual orientation could pose problems (Iran, USSR, and India), which should be considered when interpreting findings related to sexual orientation.

Research on sexual orientation also needs to consider the complexity and multidimensionality of sexual orientation, which includes three related phenomena (Wolff et al., 2017). The first, most used within biological and health sciences, is *sexual behavior*, covering sexual interactions between people of the same sex, the opposite sex, and/or both sexes (Savin-Williams, 2006). The second, most used within psychological and social sciences, is *sexual identity*, meaning the individual's self-conception as homosexual, bisexual, or heterosexual (Savin-Williams, 2006). The third phenomenon covers the individual's *sexual attraction* to the same sex, the opposite sex, or both. Bailey et al. (2016) have suggested to add a fourth aspect of sexual orientation, namely the individual's *physiological sexual arousal* to men and/or women.

It is difficult to provide exact estimates of prevalence of homosexuality and/or bisexuality due to a number of reasons (Savin-Williams, 2009; Bailey et al., 2016). For example, people who identify as heterosexual may still engage in homosexual behavior and admit homosexual attraction, without necessarily identifying themselves as homosexual (Laumann et al., 1994). Hence, findings related to sexual orientation may vary depending on the concept is measured and assessed. Second, the different phenomena associated with homosexuality and bisexuality may vary over the life course (Bailey et al., 2016), and population estimates may vary depending on whether the assessment covers the individuals' current or total lifetime history patterns of behavior and attraction. Further, which aspects of sexual orientation researchers chose to investigate will affect findings. In general, requesting information about attraction leads to higher prevalence of homosexuality than when measuring sexual behaviors (Savin-Williams, 2006). Most of the studies in this review measured sexual orientation in terms of sexual identity. If participants within these studies identified as heterosexual, but still had bi- or homosexual experiences or attractions, they would not be considered non-heterosexual. Hence, the different aspects of sexual orientation need to be taken into account when comparing findings between studies.

Another complicating factor arises in research on CAH. The terms homosexual/heterosexual are only useful if the sex of the person in question is known. However, some 46, XX individuals with CAH identify themselves as gender-fluid (de Jesus et al., 2019). In order to interpret the significance of labels like heterosexual or homosexual, the individual's self-perceived gender needs to be known. Hence, gender diversity might influence the measurement of sexual orientation, and should be taken into account in research on CAH.

### Methodological Issues

Methodological issues may have impacted some of the results that are reported in this review. First, few studies had sexual orientation as their primary outcome variable (Ehrhardt et al., 1968a; Lev-Ran, 1974; Money et al., 1984; Dittmann et al., 1992; Kuhnle et al., 1993; Meyer-Bahlburg et al., 2008; Khorashad et al., 2017), which may have affected the choice of outcome measures. In one study, sexual orientation was measured through an assessment of psychosexual development (Ehrhardt, 1979), in another as part of an assessment of feminizing genitoplasty (Gupta et al., 2006; Fagerholm et al., 2011; Lesma et al., 2014; Binet et al., 2016). In yet another study, information about registered partnerships was drawn from a national Swedish registry (Falhammar et al., 2017), and was considered an indication of sexual orientation in males with CAH. The Swedish Marriage Code became sex neutral in 2009, making it possible for individuals with non-heterosexual orientation to marry and register their partnership. However, we do not know whether all homosexual couples systematically register their partnerships. Therefore, this measure as a valid assessment of sexual orientation could be questioned. Second, most studies included individuals with CAH as a group, without providing information about subgroups (such as SW, SV, and NC or genotype). Sample characteristics (differences in included sub-groups) may therefore lead to a variation in findings. Third, when studies aimed to assess topics other than sexual orientation, concern about respondent burden may have limited the number and quality of questions regarding sexual orientation. One can also hypothesize that researchers with another primary aim for their study than the investigation of sexual orientation will put less effort into the choice of instruments, leading to a vast majority of studies using self-constructed interviews or questionnaires. Last, self-reports of an atypical sexual orientation might be susceptible to social desirability factors and may be an important source of error variance in some cultures or historical contexts (Zucker et al., 1996). Hence, results from studies conducted decades ago, or in cultural contexts where homosexuality may not be accepted or even allowed, should be interpreted with caution. Evidence derived from reports of third parties, legal registries, or parents of young children or adolescents may also be limited or in conflict with self-reports.

The current review may have missed to identify papers not overtly addressing our specific object of research. Studies presenting clinical or surgical results in CAH may have included some information about sexual orientation, without this being mentioned in the title, keywords, or abstract. The same may also be the case for articles including other conditions of sex development than CAH (DSD). If search terms, titles, keywords, or abstracts did not identify CAH as included, papers may have been missed. However, our initial search yielded 588 articles, and many of these included surgical and DSD results, and were all screened for inclusion; therefore, this review hopefully included all or at least almost all articles, dealing with sexual orientation in individuals with CAH. To facilitate the identification and inclusion of relevant studies in future reviews, researchers should consider using “sexual orientation” as a keyword, in the title, or in the abstract. Due to the large variation between conditions, studies assessing sexual orientation in groups with DSD should also consider presenting results separately for all sub-groups.

In summary, the disparity in findings may be explained by methodological differences, such as a wide range of designs, sample characteristics, concepts, and outcome measures. Other methodological challenges were associated with the large age range of participants (such as including adolescents and adults in the same sample), which may complicate an accurate assessment of sexual orientation. One of the included studies, which presented results separately for adolescents and adults, clearly illustrated possible developmental changes (Jurgensen et al., 2013), a factor that needs to be taken into account when exploring sexual orientation. Methodological challenges might in part be explained by CAH being a rare condition. Hence, large studies on homogenous samples may be difficult to conduct, but could be solved by multicenter and international studies including larger number of participants.

### Clinical Implications

The current review indicates that females with CAH more often than the general population define themselves as non-heterosexual, which could have clinical implications. Other research findings pertaining research on CAH have shown psychologic and psychiatric problems to be more frequent in this group, particularly anxiety or depressive disorders and drug/alcohol abuse (Hochberg et al., 1987; Falhammar et al., 2014a; Engberg et al., 2015; Pasterski et al., 2015; Daae et al., 2018; Khorashad et al., 2018). In addition, some females with CAH will go through feminizing surgery (Crouch et al., 2008) and/or may show symptoms of androgen excess, e.g., hirsutism (Meyer-Bahlburg et al., 2018). Some could also experience stigma or fear of rejection in romantic and/or sexual situations, due to genital and non-genital physical features caused by CAH (Meyer-Bahlburg et al., 2018). Awareness of having a functionally inadequate vagina and of experiencing reduced erotic sensitivity and orgasmic capacity might also reduce sexual interest (Meyer-Bahlburg, 1999), and clinicians should be aware of potential body image problems because of short stature, lack of breast development, or hirsutism. Females with CAH may therefore be exposed to more risk factors than the general population, which might in turn affect sexuality. Clinicians should explore whether and to what extent such factors may affect the patient psychologically.

Some individuals may feel vulnerable and uncomfortable talking about sexual orientation, and clinicians should be aware of concepts used, so that respect and understanding is communicated. When indicated and if relevant, sexual orientation should be assessed by addressing all dimensions of sexual orientation: identity, attraction, and behavior. Clinicians can be in a unique position to help their patients sort out thoughts and feelings concerning this delicate issue, identify whether an individual's sexual orientation is experienced as subjectively problematic, and if necessary, refer the patient to adequate support and intervention.

Research indicates that sexual minorities may be overrepresented in clinical settings, due to higher suicidal rates, anxiety and mood disorders compared with their heterosexual peers (Herek and Garnets, 2007; Mustanski et al., 2010), and non-heterosexual youth also tend to engage more in health-risk behaviors like illegal substance abuse, unhealthy weight control practices, and risky sexual behaviors than heterosexual youth (Garofalo et al., 1998). Moreover, experiences of discrimination may occur in employment, education, and health care, but also in meaningful interpersonal relationships such as family (Milburn et al., 2006; Feinstein et al., 2014; António and Moleiro, 2015). Hence, identifying individuals in need of psychological follow-up is central. Nevertheless, clinicians also need to be open for a potential positive impact of non-heterosexual orientation. Although challenges faced by non-heterosexuals has been well documented, strength and resiliency, and other positive aspects of being homosexual have also been demonstrated in the literature (Harper et al., 2012).

## Conclusion

Females with CAH seem less likely to have an exclusively heterosexual orientation than females from the general population, whereas no males with CAH identified themselves as non-heterosexual in the present review. However, the interpretation of findings need to take into account the wide diversity in measures used for assessing sexual orientation in individuals with CAH, and a preference for unvalidated and self-constructed interviews and questionnaires that complicates conclusions and impede comparisons between studies. Hence, methodological issue may have led to the number of individuals with non-heterosexual orientation to be overestimated or underestimated. The methodological challenges that are identified in the current review should be addressed in future research.

## Data Availability Statement

The raw data supporting the conclusions of this article will be made available by the authors, without undue reservation, to any qualified researcher.

## Author Contributions

ED: conceptualization, methodology, validation, formal analysis, investigation, resources, writing original draft, project administration, writing review and editing, and visualization. KF: conceptualization, methodology, validation, formal analysis, investigation, resources, writing original draft, writing review and editing, visualization, supervision, and project administration. AW: validation, investigation, writing original draft, writing review and editing, and visualization. IN: validation, investigation, resources, writing original draft, writing review and editing, and visualization. HF: conceptualization, methodology, validation, investigation, resources, writing original draft, writing review and editing, visualization, supervision, and project administration.

### Conflict of Interest

The authors declare that the research was conducted in the absence of any commercial or financial relationships that could be construed as a potential conflict of interest.
